# Calcific Aortic Valve Disease: a Developmental Biology Perspective

**DOI:** 10.1007/s11886-018-0968-9

**Published:** 2018-03-08

**Authors:** Punashi Dutta, Joy Lincoln

**Affiliations:** 1 Center for Cardiovascular Research, The Research Institute at Nationwide Children’s Hospital, 575 Children’s Drive, WB4239, Columbus, OH 43215 USA; 2 The Heart Center, Nationwide Children’s Hospital, Columbus, OH USA; 3 Department of Pediatrics, The Ohio State University, Columbus, OH USA

**Keywords:** Heart valve, Calcification, Valvulogenesis, Cell signaling, Extracellular matrix

## Abstract

**Purpose of Review:**

This review aims to highlight the past and more current literature related to the multifaceted pathogenic programs that contribute to calcific aortic valve disease (CAVD) with a focus on the contribution of developmental programs.

**Recent Findings:**

Calcification of the aortic valve is an active process characterized by calcific nodule formation on the aortic surface leading to a less supple and more stiffened cusp, thereby limiting movement and causing clinical stenosis. The mechanisms underlying these pathogenic changes are largely unknown, but emerging studies have suggested that signaling pathways common to valvulogenesis and bone development play significant roles and include Transforming Growth Factor-β (TGF-β), bone morphogenetic protein (BMP), Wnt, Notch, and Sox9.

**Summary:**

This comprehensive review of the literature highlights the complex nature of CAVD but concurrently identifies key regulators that can be targeted in the development of mechanistic-based therapies beyond surgical intervention to improve patient outcome.

## Introduction

Calcific aortic valve disease (CAVD) is a public health problem affecting up to 13% of the population over the age of 65, and prevalence increases in the general population as the median age rises [1]. Twenty five percent of people aged over 65 have a 50% increased risk of cardiovascular related events, and if untreated, there is an associated risk of 80% over 5 years of progression to heart failure or death [2]. This common cardiovascular disorder is characterized by an abnormal accumulation of calcium-rich nodules on the aortic surface and/or within the annular region of the valve cusp, leading to thickening termed *sclerosis*, limited movement, and stenosis (left ventricular outflow obstruction) [3]. At present, surgical valve replacement remains the standard treatment option which comes with insuperable complications, financial burdens, and no guarantee of long-term success. Furthermore, there are no approved pharmacological treatments available to stop the progression or treat (reverse) CAVD. Therefore, there is an increasing critical need to develop new medical therapies.

The underlying etiology of CAVD is poorly understood, but clinical risk factors have been identified; many of these are common to other cardiovascular disorders including atherosclerosis. Elevated total cholesterol, low-density lipoprotein (LDL) triglycerides, decreased high-density lipoproteins, male sex, tobacco use, hypertension, and diabetes mellitus have been reported to increase the incidence of aortic stenosis [4, 5]. It is not yet clear how these *environmental* risk factors promote CAVD onset, but in atherosclerosis, infiltration of inflammatory response cells and endothelial cell dysfunction (oxidative stress) are associated [6, 7•]. In addition to these factors, there are reports of a genetic component with mutations in *Notch1* being identified [8]. Two percent of the population is born with bicuspid aortic valve (BAV), and approximately 50% of these patients will develop CAVD at an earlier age than individuals with tricuspid aortic valves [9, 10]. This premature onset is thought to be attributed to changes in the biomechanical environment and the abnormal mechanical stress elicited by the morphological defect. While this has been the accepted dogma in the field, the cause of abnormal mechanical stress in affected patients is largely unknown, and the mechanosensory pathways that promote calcific changes in susceptible patients have not been identified. This review provides a concise overview of the current literature related to the importance of structure-function relationships in healthy valves and the key molecular players that contribute to their formation. In addition, we discuss the re-activation of valvulogenesis and bone development signaling pathways in the onset and progression of CAVD.

## Structure-Function Relations of Healthy Aortic Heart Valves

Heart valves are dynamic structures opening and closing over 100,000 times a day to regulate unidirectional blood flow from the left ventricle to the rest of the body. There are two sets of cardiac valves: the atrioventricular (AV) valves, including the mitral and tricuspid that separate the atria from the ventricles, and the aortic and pulmonic semilunar valves that separate the ventricles from the great arteries. Although the functional demand of each valve set is similar, their anatomies are different. The AV valves are situated in the atrioventricular canal separating the atria from the ventricles. Structurally, these valves consist of two (mitral) or three (tricuspid) leaflets, with external supporting chordae tendineae that attach the leaflet to papillary muscles within the ventricles (reviewed in [11]). In contrast, the semilunar valves located at the base of the aorta and pulmonary trunk are comprised of three leaflets termed *cusps* and lack external chordae and papillary muscles, although a unique internal support structure has been described [12]. It is the coordinated movement of these dynamic valvular structures that maintain unidirectional blood flow during the cardiac cycle. In diastole, the papillary muscles are relaxed and high pressure in the atrium causes opening of the mitral (left) and tricuspid (right) valve leaflets to promote blood flow into the respective ventricle. Once ventricular pressure increases during diastole, the chordae *pull* the atrioventricular valve leaflets closed and maintain coaptation to prevent eversion of the valve into the atria. As the ventricle contracts, blood exits through the now open semilunar valves and the ventricle relaxes to begin the cycle again. Therefore, throughout the cardiac cycle, the heart valve structures are exposed to constant changes in hemodynamic force as a result of pressure differences between systole to diastole. To withstand this complex mechanical environment, the valve leaflets/cusps develop and maintain an intricate and highly ordered connective tissue system [11].

Opening and closing of the valve leaflets or cusps is largely achieved by three organized layers of extracellular matrix (ECM) arranged according to blood flow direction, that each provide a unique biomechanical property to withstand the complex hemodynamics experienced with every cardiac cycle [12]. The fibrosa layer is situated furthest away from blood flow and is largely composed of bundles of aligned fibrillar collagens that provide strength. Organized elastic fibers make up the ventricularis/atrialis (semilunar/atrioventricular) layer situated adjacent to blood flow. This matrix component allows for valve extension and recoil during each heartbeat [13]. The proteoglycan-rich spongiosa layer is *sandwiched* between the fibrosa and ventricularis and provides compressibility in these load-bearing regions [14].

**Fig. 1:**
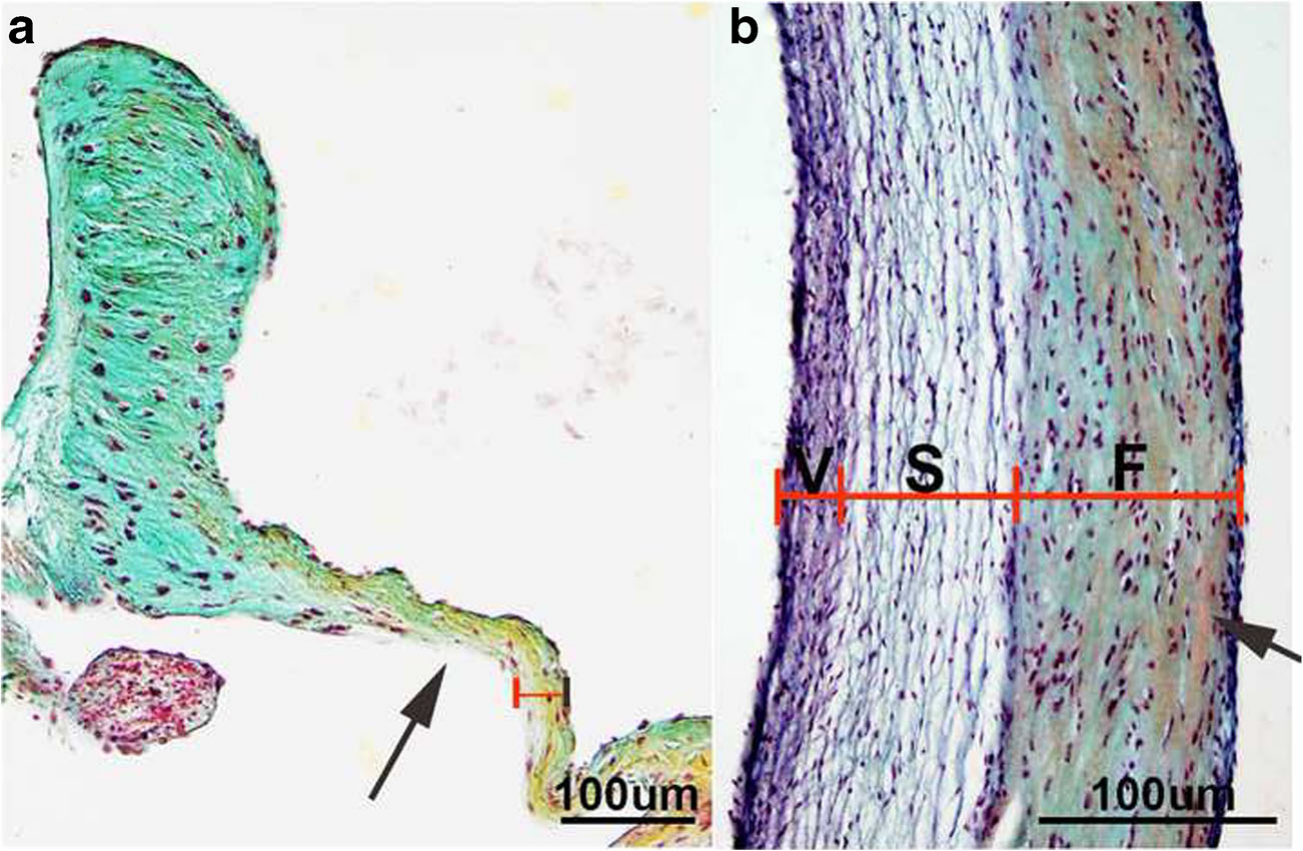
Overview of conserved mature heart valve structure. Pentachrome staining to show extracellular matrix organization within the aortic valve structure of mice (**a**) and sheep (**b**). Note more defined stratification in the larger animal model. F = Fibrosa S = Spongiosa V = Ventricularis

The overall valve structure, matrix composition, and organization are conserved across many species with more apparent order being observed in larger animals (see Fig. 1) [12]. Interestingly, there are exceptions presumably due to differences in physiological demand. For example, the tricuspid valve of the avian species is largely composed of myocardial tissue and this may be attributed, in part, to variation in size and hemodynamic burden [15]. Alligators and crocodilians have a cog-wheel valve, and this differential structure supports the anatomical design that consists of left and right ventricles that directly connect to the great vessels [16]. Furthermore, in a comparative study, it was found that the aortic valves in giraffes are significantly stiffer than those in bovine due to increased elastin content and more compact collagen, which likely favors their naturally high blood pressure which is twice that of humans [17]. These collective studies highlight the importance of the valve structure and composition for adaptive function throughout life.

Homeostasis of the valve ECM is maintained by a heterogeneous population of valve interstitial cells (VICs) that, in healthy adults, are phenotypically similar to fibroblasts and express vimentin [18]. The VICs largely serve to mediate physiological ECM remodeling within the leaflet/cusp in response to the normal *wear and tear* of aging. This is achieved through a balanced secretion of matrix degradation enzymes, including matrix metalloproteinases (MMPs) and their inhibitors (TIMPs), and deposition of structural matrix components within the three layers [19, 20]. Therefore, the VIC population plays a critical role in preserving the architecture of the valve for functional biomechanics. In addition to this cell population, the valve leaflet or cusp is encapsulated by a single cell layer of valve endothelial cells (VECs) that primarily functions as a barrier between the blood and the inner valve tissue, thereby protecting against the physical mechanical stress of the hemodynamic environment and preventing excess infiltration of circulating risk factors and inflammatory cells [21, 22]. In addition, VECs have been shown to molecularly communicate with underlying VICs to regulate their phenotype [23, 24]. VEC-specific disruption of essential signaling pathways in mice can alter VIC function and ECM organization leading to dysfunction [23, 25–28]. Therefore, the integrity and function of the valve endothelium appears to be essential for maintaining structure-function relationships throughout life.

## Aortic Valve Development

Formation of the highly ordered mature valve structure begins during embryonic (E) stages, around E9.5 in the mouse and E27 in humans. Prior to septation, the looped heart predominantly consists of cardiac myocytes and an overlying layer of endocardial cells that are separated by a hyaluronan-rich matrix, termed *cardiac jelly*. Specific to the atrioventricular canal and outflow tract regions, a subset of endocardial cells undergoes endocardial-to-mesenchymal transformation (EMT) and gives rise to *swellings* known as endocardial cushions, composed of mesenchymal cells embedded within a hyaluronan-rich ECM. Previous lineage tracing studies using endocardium-specific *Cre* mouse lines demonstrate that endothelially derived mesenchymal cells serve as precursors to the mature valve structures [29, 30]. The process of EMT is initiated in response to signals largely emanating from the adjacent myocardium and predominantly includes transforming growth factor-β (TGF-β) signaling. Studies in chick indicate important roles for the ligands TGF-β2 and TGF-β3 in early initiation steps [31–33], while in mice, endocardium-specific deletion of canonical Wnt signaling (β-catenin dependent) inhibits TGF-β-mediated induction of EMT, suggesting crosstalk between these signaling pathways [34]. Bone morphogenetic protein (BMP) receptors and ligands are another major source of myocardially derived signals for EMT initiation [32, 35–42]. Myocardium-specific knockdown of *BMP2* severely disrupts VEC transformation, particularly in the atrioventricular canal, suggesting specificity to this valvular position [43]. Furthermore, Notch signaling, predominantly in VECs, is a potent activator of EMT and roles for both receptors and ligands have been identified [27, 44–51].

Following initiation of EMT, newly transformed mesenchymal cells then migrate and proliferate within expanded cardiac jelly and give rise to swellings known as endocardial cushions [33, 52]. In mice lacking *TGF-β2*, EMT is initiated; however, cushions are variably hypoplastic as a result of defects in migration and/or proliferation [53, 54], therefore suggesting differential roles for TGF-β signaling during cushion formation. Similar cushion defects are also observed in other mouse models with targeted downregulation of TGF-β signaling including endocardial deletion of *TGF-βRI* (*Alk5*) [55] and global deletion of the long form of latent TGF-β-binding protein 1, in which EMT initiation is impaired [56]. In addition to TGF-β signaling, the transcription factor Sox9 has been shown to be a key player of proliferation of newly transformed mesenchymal cells. Targeted loss of *Sox9* function using the endocardial *Tie2cre* line results in premature lethality around E11.5 due to a failure to expand the valve precursor cell pool within the endocardial cushions [57]. The temporal window of EMT is somewhat diffuse, and while many positive regulators have been reported, few regulators to stop the process are known. Fate mapping using a valve endothelium-specific *Cre* mouse line shows that cells expressing a Nfatc1-enhancing region do not undergo EMT and remain in the endothelium [58]. Therefore, EMT is a *finely tuned* process and studies in mice highlight the critical need to balance positive and negative regulators as genetically altered mice with severe cushion defects suffer premature lethality (reviewed in [11]).

The contribution of endocardially derived cells to the aortic valve precursor cell pool in the outflow tract and atrioventricular canal region was first demonstrated using the *Tie2cre*;*Rosa26R* reporter model, although it was noted that not all precursor cells recombine with the *Tie2cre* transgene [29, 30]. Following these studies, it was later shown that cells originating from the neural crest (*Wnt1cre*) and second heart field (*Mef2ccre*) also contribute to the semilunar valve precursor cell pool [59–61]. Interestingly, significantly less neural crest and secondary heart field-derived cells contribute to the mitral and tricuspid positions. However, these valves, unlike the semilunar valves, receive WT1-positive cells derived from the adjacent epicardium [62]. While these developmental studies are informative, the field has yet to delineate the purpose or function of differential cell lineage contributions to the primitive atrioventricular and semilunar valve structures.

Once the valve precursor pool of mesenchymal cells has been established (around E14.5 in the mouse), the endocardial cushions undergo extensive remodeling as they elongate and thin into primordia. Cell proliferation is significantly reduced at this time, although proliferative cells remain enriched at the tip [12]. Concurrently, precursor cells loose mesenchymal molecular markers including Twist1 but gain the activated myofibroblast marker, α-smooth muscle actin (α-SMA) [18]. This phenotypic change is thought to reflect transition towards an activated valve interstitial cell (aVIC) that mediates physiological remodeling of the ECM during this stage of maturation. This includes breakdown of primitive cardiac jelly and synthesis of new ECM components that will later form the fibrosa, spongiosa, and ventricularis layers. However, direct evidence of embryonic aVIC function is lacking. The molecular regulators of mid-to-late valve development are largely unknown, but pathways important for EMT including TGF-β, BMP, Wnt, and Sox9 are also active during remodeling and have been shown to play differential roles at this stage (Gallina and Lincoln, unpublished) [57, 63–65]. More recently, additional regulators have been reported including hypoxia, cadherin-11 (cell adhesion), the chemokine receptor CXCR7, and the matrix remodeling enzyme ADAM17 [66–69]. While mouse models with targeted genetic disruptions that result in valve remodeling defects are viable, it is considered that defects at this stage could underlie congenital valve malformations present at birth or potentially acquired disease manifested later in life.

The primitive valve continues to grow and mature after birth, and in the mouse, the three layers of predominant ECM components (collagen, proteoglycan, elastin) are apparent between postnatal days (PNDs) 7 and 10. At this time, cell proliferation is around 16.3% in VECs and ~ 15.2% in VICs (based on the 7-h pulse change of EdU) and cell division remains at this frequency until around PND 4 [12, 22] (Nordquist and Lincoln, unpublished). Concurrently, VICs lose α-SMA but maintain vimentin expression, suggesting transition towards a quiescent (or non-activated) fibroblast-like cell type [18]. This quiescent phenotype is maintained throughout life in the absence of disease with cell proliferation estimated at a lower frequency of ~ 2.0% in VECs and ~ 1.1% in VICs (7-h pulse chase) [22]. This level of normal adult cell turnover in the valve might be considered high compared to other cardiac cell types (< 1% in cardiac myocytes) [70]; however, the overall valve cell number does not appear to increase with aging (but matrix synthesis does) and, therefore, cell death likely occurs at a similar frequency; however, further studies are needed to determine this. The mechanism for maintaining adult valve cell population during the normal wear and tear of aging relies not only on resident cell proliferation but also on the contribution of extracardiac cells. Using mouse models to fate map CD45-positive cells, we and others have shown that under homeostatic conditions, ~ 2.3% of the valve cell population is derived from this lineage at postnatal stages and up to 10.3% at 6 weeks [71–74]. It is speculated that loss or gain of VEC and VIC number might lead to perturbations in ECM homeostasis and subsequent biomechanical defects. In addition, the function of these cells is also important. In many cardiovascular disorders, endothelial cell dysfunction has been shown to play a major role. In the valves, we have shown that VECs have an overall decrease in nitric oxide availability, metabolism, membrane self-repair, and endothelial-to-mesenchymal transition potential with aging [22]. Furthermore, studies have reported an age-associated loss in ECM organization and hemostatic protein regulation [75, 76], therefore suggesting that physiological regulators of valve homeostasis are sufficient to maintain structure-function relationships until the age of ~ 65 in otherwise healthy subjects or earlier in patients carrying known risk factors.

## Calcific Aortic Valve Disease

CAVD is the most predominant form of valve pathology affecting more than 5.2 million people in the USA, particularly those over the age of 65 [77]. In 2013, 50,222 deaths occurred due to valvular heart diseases in the USA, out of which 67.5% were due to aortic valve disorders [78]. Traditionally, CAVD was seen as a degenerative process, as a result of aging of the aortic valve. However, several lines of evidence suggest that CAVD is an active disease with discernible initiating factors, including clinical and genetic predisposition, and dysregulation of molecular and cellular pathways that facilitate disease progression [79]. Many of these factors are thought to be shared with atherosclerotic plaque formation and vascular calcification; however, parallels in pathogenic mechanisms remain elusive but warrant further investigation. At present, effective pharmacological treatments are lacking and interventional surgery or procedures to replace calcified or stenotic valves are the only effective option with no long-life guarantee [80]. This clinical limitation has been largely attributed to our lack in understanding of CAVD pathogenesis. However, the field is growing and the mechanisms underlying disease onset and progression are emerging.

Calcification of the aortic valve is characterized by overall thickening of the valve cusp and the presence of calcium-rich nodules on the aortic valve surface and/or within the annulus region, leading to functional stiffening and stenosis [81]. CAVD is slow and progressive, and in human pathology, early stages are associated with (i) endothelial dysfunction as indicated by oxidative stress following exposure to known risk factors (aging, high LDL levels, etc.) [22, 82, 83]. Worthy of mention, oxidative stress is the standard measure of endothelial cell dysfunction in diseased valves, but recent reports from our group have identified additional parameters that should be considered when defining this pathogenic phenotype [22]; and (ii) inflammation leading to infiltration of immune cells including T cells and monocyte-derived macrophages [84, 85], which could be the result of endothelial dysfunction and failure to maintain the physical barrier between the inner cusp and circulating blood. Collectively, these abnormalities in endothelial cell function, other cell contribution, and likely other currently unknown mechanisms trigger pro-osteogenic processes. It remains unclear how known risk factors progressively lead to calcific nodule formation; however, genetic manipulation studies in mice, often with added risk factors including diet, have identified key regulators that contribute at some stage of the pathogenic program and these are summarized in Table 1. In addition to in vivo models, many groups have developed in vitro assays to study the mechanisms of CAVD. The most well-established protocol involves culturing VICs in osteogenic media (ascorbic acid, β-glycerophosphate, and dexamethasone) to stimulate calcific nodule formation. In addition, others have supplemented media with inorganic phosphate (sodium phosphate dibasic), mimicking hyperphosphatemia in chronic kidney disease largely associated with increased CAVD [86, 87]. We and others have shown that altered biomechanics can also promote calcific nodule formation when VICs are cultured on stiff matrices such as glass [24, 88, 89], or tissue culture polystyrene pre-coated with fibrin, laminin, and heparin also leads to an increase in the number of calcific nodules [90] or the addition of TGF-β [91]. In most assays, investigators utilize VICs isolated from human, porcine, and ovine models as these have been previously reported to have potential to undergo calcification in vitro (reviewed in [92]). Similar protocols for murine VICs have been more technically challenging, although intact, whole aortic valve explants can undergo osteogenic changes upon stimulation [93, 94]. Interestingly, rat VICs exhibit comparatively low calcification potential (as indicated by Alizarin Red staining) but do express pro-osteogenic molecular profiles when stimulated [95], suggesting a species-dependent limitation to reach end-stage calcium deposition.

**Table 1:** Published mouse models of calcific aortic valve disease

Mouse model	Associated human disease	Valve phenotype	References
Apoe^−/−^ Apoe^−/−^ on atherogenic diet	AoV calcification	Thickened AoV, calcification, AS thickened AoV	[96, 97]
Chm1^−/−^		Thickened AoV, calcification	[98]
B6-Egfr^wa2/wa2^ Egfr^wa2/wa2^:Ptpn11^+/−^	AoV hyperplasia Valve thickening	Thickened AoV, calcification Thickened AoV and PV	[99, 100]
Klotho null	AoV calcification	AoV calcification	[101]
LDLR^−/−^;Apob^100/100^		AoV calcification	[102]
NOS3^−/−^;Notch1^+/−^ fed Western diet	BAV, AoV calcification	AoV calcification, BAV	[23]
Notch1^+/−^ on Western diet	BAV and AoV calcification	Thickened and calcified AoV	[103]
Sox9^+/−^;Col2a1-Cre	AoV calcification	AoV calcification	[57, 94]
RBPJk1^+/−^ fed HCVD diet	BAV, AoV calcification	Thickened AoV, calcification	[104, 105]
RBP-J^f/f^-MxCre (RBPKO)		Enlarged AoV	
C57BL/6J fed excess vitamin A diet	AoV calcification	AoV calcification	[106]
VDR^−/−^ fed Western diet	AoV calcification	AoV calcification	[107]
LDLR^−/−^ fed Western diet with low vitamin D			

*AoV* aortic valve; *PV* pulmonary valve; *BAV* bicuspid aortic valve; *HCVD* high cholesterolemic and vitamin D supplement

As discussed, the mechanisms that promote abnormal pro-osteogenic changes in valvular structures following exposure to risk factors or genetic predispositions are largely unknown. However, there are several reports that shed light on the biological processes that might be involved. One theory is that in response to pathological stimuli, resident VICs become *activated* as identified by positive α-SMA staining and transdifferentiate towards an osteoblast-like lineage. This is associated with abnormal activation of signaling pathways common to valve and bone development and expression of molecular markers observed in mineralized tissue, including *Runx2*, *osteopontin*, *osteocalcin*, *bone sialoprotein*, *matrix Gla protein*, and others [108, 109]. Although CAVD and bone mineralization share common mediators, they are quite different anatomically based on crystal size, mineral morphology, and elemental composition [110].

Several signaling pathways and transcription factors involved in endocardial cushion formation and bone development are reported to be dysregulated in CAVD. It remains unclear if abnormalities in these developmental regulators *cause* pro-osteogenic changes or are the *effect* of CAVD. Such pathways include TGF-β, implicated in the positive regulation of α-SMA during early VIC activation [111]. However, it should be mentioned that the requirement of VIC activation for subsequent pro-osteogenic differentiation of VICs has not been directly tested. Explanted human aortic valves from CAVD patients with end-stage disease show increased expression of TGF-β1, and TGF-β1 treatment of ovine VICs is sufficient to promote calcification when cultured in osteogenic media, suggesting positive regulation [112, 113]. Similarly in developing bone, TGF-βs are pro-osteogenic, although it is the *TGF-β2*, but not *TGF-β1* or *TGF-β3*, that is critical for inducing osteogenesis in mice [53, 114–116]. In contrast to this role, our group showed that TGF-β1 treatment of porcine VICs cultured on glass to promote pro-osteogenic changes prevented calcific nodule formation, and deletion of *TGF-β1* in VECs causes CAVD in mice [25]. These findings suggest that TGF-β plays pivotal roles in calcification which could be dependent on differential concentrations of endogenous or exogenous ligands, as previously described in osteoblast systems (reviewed in [117]).

The BMP family is named accordingly due to their requirement for bone formation. BMP2 is the major inducer of bone formation, but other ligands such as BMP7 may also mediate the osteogenic response through BMPR1A/ALK3, BMPR1B/ALK6, and AcvR1/ALK2 receptors [118–128]. Studies of calcified human valves show increased *BMP2* and *BMP4* expression and the expression of canonical BMP signaling mediator, pSmad1/5/8 [129–131]. The direct contribution of activated BMP signaling to CAVD pathogenesis has not been extensively examined. However, inhibition of BMP signaling by genetic inactivation of *BMPR1A* prevents CAVD in a susceptible mouse model (*Klotho*^*−/−*^), and tissue-specific deletion of the *Acvr1/ALK2* receptor leads to bicuspid aortic valve in mice and enhance pro-osteogenic changes during adulthood [132]. Hence, *BMPR1A* is required for the valvular calcification while Acvr1 prevents BAV and subsequent nodule formation.

Canonical Wnt signaling (β-catenin dependent) is increased in calcified valves from human patients, mouse models, and cultured VICs [133–138]. As with TGF-β signaling, it is not known if increased Wnt underlies the cause of CAVD or is a read-out of the end-stage process. Wnt activation promotes VIC activation [81] and, in other systems, promotes osteogenic differentiation of progenitor cells [139, 140] and induces calcification of vascular smooth muscle cells through β-catenin-mediated regulation of *Runx2* [141]. At present, the direct contribution of Wnt signaling to CAVD remains unclear, but these studies warrant further investigation.

As shown in Table 1, genetic alterations in Notch signaling family members promote CAVD in mice and human patients with *Notch1* mutations correlate with aortic valve disease including calcification [8]. Several groups have shed light on the mechanisms underlying the role of endothelially derived Notch1 receptor in calcification, and these include positive regulation of osteogenic inhibitors including matrix gla protein and *Sox9* [95, 142]. At the level of the ligand, deletion of *Jag1* in endothelially derived cells in mice leads to calcification associated with valve development defects [143]. Notch activation favors BMP-induced osteoblast differentiation during skeletal development, and *Notch* inhibition represses BMP target genes [144–146]. Furthermore, BMP2 and TGF-β regulate expression of Notch pathway signaling proteins [147]. Together, these findings highlight the crosstalk between Notch and osteogenic signaling pathways.

In addition to its role in valvulogenesis, Sox9 is an important mediator of CAVD. Initial studies of skeletal development identified *Sox9* as a critical regulator of cartilage formation. In mouse chimeras, *Sox9*^*−/−*^ cells are excluded from all cartilage tissues and fail to express chondrocyte-specific markers [148]. In addition, *Sox9* haploinsufficiency leads to defective cartilage primordia and premature skeletal mineralization [149]. The mechanisms of this inhibitory role for Sox9 in bone formation remain largely unknown, but Sox9 is known to inhibit Runx2 function at the protein level and directly represses its transactivation function on target genes [150, 151]. In the valves, we showed that reduced function of *Sox9* during mid-stages of valvulogenesis is sufficient to promote early-onset CAVD in mice [57, 94], and this was mediated, in part, through de-repression of the osteogenic matrix protein *Spp1* [93]. The transcriptional activity of Sox9 on *Runx2*, *Spp1*, and potentially, other markers requires its nuclear localization. We have shown that reduced nuclear Sox9 precedes calcification of both mouse valves in vivo and pAVICS in vitro, and this is likely modulated through paracrine signals emanating from overlying VECs including TGF-β1, as well as regulation via nuclear export and import signals [24] (Dutta and Lincoln, unpublished).

Studies have shown that in parallel with pro-osteogenic fate changes in VICs during the development of CAVD, a significant contribution by extracellular vesicles (EVs) to the formation of calcific nodules is found and is localized at sites within the valve structure. EVs are membrane vesicles that are secreted from cells containing intracellular contents [152]. They encompass a broad range of vesicles with varied features. Major types of EVs include microvesicles which are released from the budding of plasma membrane, and exosomes which originate from endosomes [152]. The evidence of their presence has been validated via ultrastructural analysis and is found to be localized within medial arterial calcifications, atherosclerotic intimal plaques, and calcified human aortic valves [153]. EVs serve as nucleating foci for calcific mineral crystallization via interacting with fibrillar collagen; however, the mechanism of this interaction and initiation of micro/macrocalcification remains unclear [153]. Specific to aortic valve calcification and following endothelial cell dysfunction of diseased valves, EVs are thought to be derived from inflammatory cells including leukocytes, platelets, and endothelial cells themselves. These EVs promote VIC activation and subsequent fibrosis and mineralization [152]. The molecular regulation of EVs in calcification is not well known, but recently, Aikawa’s group reported a novel role for the glycoprotein sortilin, which resides in calcifying vessels in human and mouse atheromata. Sortilin contributes to the formation of microcalcifications in smooth muscle cell culture through interaction with caveolin-1 and tissue non-specific alkaline phosphatase [154•]. While EVs have been observed in CAVD, the presence of vascular regulators remains unclear.

As discussed, there is a growing interest in discovering new molecular pathways in CAVD pathogenesis and the contribution of mechanical stress and flow to disease onset and progression continues to be an active area of investigation among biomedical engineering groups. The mechanical stimuli experienced by the aortic valve include shear stress strain, and pressure that alter strain/stress in the leaflet tissue [155]. The aortic valve largely faces two kinds of stress: oscillatory flow or shear stress on the fibrosa side of the valve and laminar on the ventricularis side [155]. The fibrosa layer of the valve predominantly experiences oscillatory shear, and this region is more prone to calcification than the ventricularis area that senses laminar shear. Simmons’ group has previously shown that endothelial cells on the fibrosa side express pro-osteogenic mediators while cells on the ventricularis surface express calcification inhibitors [156]. However, it is not clear if the differential biomechanical environment, the diversity between VEC populations and location, and potentially, the development origin influence the profile of their mRNA expression. While the observation of oscillatory and laminar flow patterns has been known for many years, Dasi’s group more recently showed patient-specific differences in aorta anatomy that lead to differential flow patterns associated with calcific nodule formation at localized sites [157]. These findings may help to explain the increased susceptibility in certain individuals including those with BAV that experience a drastic change in flow momentum caused by the eccentricity of the orifice jet as a result of two leaflets instead of three. More specific to strain, Merryman’s group has shown that subjecting VICs to strain using the Flexcell tension system following TGF-β1 treatment augments calcific nodule formed compared to no strain (with TGF-β1 treatment) [158]. In addition, the positive contribution of mechanical strain to calcification has more recently been shown using the finite element method [159].

In conclusion, it remains unclear whether mechanical stress initiates pro-osteogenic changes or subtle changes in valve morphology and stiffness that lead to changes in flow pattern. Several decades ago, the fields of valve biology and valve bioengineering were distinct entities. Today, however, these two fields have been highly integrated in recent years in order to fully understand the multifaceted process of CAVD. While this is advantageous to those elucidating pathogenesis, the complexity of the biology makes the development of an alternative therapy challenging.

## Clinical Management

The management of CAVD has received increased attention over the last decade due to an increase in disease prevalence as the global population lives longer [160, 161]. In addition, there is a significant cohort of patients with severe symptomatic aortic stenosis that are left untreated, and others that develop left ventricular dysfunction before the onset of detectable aortic valve regurgitation [5, 80, 162, 163]. While the field works towards identifying biomarkers of CAVD and discovering new pharmacological-based treatment strategies, conventional surgery to replace the calcified and dysfunctional aortic valves remains the gold standard intervention for patients. However, data from large registries still show a mortality rate of 2–3% and this risk is increased in patients with comorbidities [164–166]. Mechanical and bioprosthetic valves are generally available for valve replacement surgeries. Mechanical valves are rigid and largely free from structural failure and therefore common in younger (aged under 50) CAVD patients with *normal* hemodynamics. However, due to the material composite, mechanical valves fail to remodel in the patient and are thrombogenic, requiring life-long anticoagulation therapy [80]. The use of biological replacement valves has increased from 43.6% in 1997 to 78.4% in 2006 [167]. These are the most commonly implanted xenograft material made from a native porcine valve or root or from bovine pericardium. Long-term anticoagulation is not required, and the hemodynamics is superior. However, unlike the mechanical valve, biological valves are prone to degenerative wear and tear like the native valve. In summary, the implantation of prosthetic aortic valves is not a perfect solution to CAVD and the field is in critical need of more effective alternatives.

Pharmacological initiatives in the treatment of CAVD have focused on targeting risk factors of CAVD in affected individuals, particularly elevated lipoprotein levels. The outcomes of administering oral statin therapy to lower LDL levels have been mixed. Despite some studies initially reporting benefits [168–173], others have shown conflicting results, with some stating no beneficial effects on valve structure and function despite a significant drop in LDL cholesterol levels [174–181]. Therefore, statin therapy for CAVD remains underprescribed and the field continues to explore better alternatives. Unfortunately, none to date has been found to work better than prosthetic valve replacement, which is likely due to the complex, multifactorial nature of this disease and the difficulty in targeting multiple contributing signaling pathways discussed in this review.

## Conclusions

The pathogenesis of CAVD is complex, and its manifestations appear later in life likely resulting from long-term exposure to known risk factors, leading to altered biomechanics and re-activation of signaling pathways important in developing valves and bone. In addition to acquired diseases, it is also important to consider that CAVD in the elderly may have origins during valvulogenesis that increase susceptibility to changes after birth. The clinical management of CAVD patients is ever changing with advancements in imaging capabilities to improve diagnosis; however, treatment options remain a challenge and non-invasive approaches are limited. By gaining a more complete understanding of the molecular and cellular processes that prevent calcification in healthy valves and promote osteogenic-like changes in at-risk valves, we can move forward in developing alternative, mechanistic-based therapies to improve patient long-term outcome.

## References

[1] Nasir K, Katz R, Takasu J, Shavelle DM, Detrano R, Lima JA (2008). Ethnic differences between extra-coronary measures on cardiac computed tomography: multi-ethnic study of atherosclerosis (MESA). Atherosclerosis.

[2] Otto CM, Burwash IG, Legget ME, Munt BI, Fujioka M, Healy NL (1997). Prospective study of asymptomatic valvular aortic stenosis. Clinical, echocardiographic, and exercise predictors of outcome. Circulation.

[3] Otto CM, Prendergast B (2014). Aortic-valve stenosis—from patients at risk to severe valve obstruction. New Engl J Med.

[4] Mohler ER (2000). Are atherosclerotic processes involved in aortic-valve calcification?. Lancet.

[5] Stewart BF, Siscovick D, Lind BK, Gardin JM, Gottdiener JS, Smith VE (1997). Clinical factors associated with calcific aortic valve disease. Cardiovascular Health Study. J Am Coll Cardiol.

[6] Yang X, Li Y, Li Y, Ren X, Zhang X, Hu D (2017). Oxidative stress-mediated atherosclerosis: mechanisms and therapies. Front Physiol.

[7] Gimbrone MA, Garcia-Cardena G (2016). Endothelial cell dysfunction and the pathobiology of atherosclerosis. Circ Res.

[8] Garg V, Muth AN, Ransom JF, Schluterman MK, Barnes R, King IN (2005). Mutations in NOTCH1 cause aortic valve disease. Nature.

[9] Roberts WC, Ko JM (2005). Frequency by decades of unicuspid, bicuspid, and tricuspid aortic valves in adults having isolated aortic valve replacement for aortic stenosis, with or without associated aortic regurgitation. Circulation.

[10] Aksoy O, Cam A, Agarwal S, Ige M, Yousefzai R, Singh D (2014). Significance of aortic valve calcification in patients with low-gradient low-flow aortic stenosis. Clin Cardiol.

[11] Tao G, Kotick JD, Lincoln J (2012). Heart valve development, maintenance, and disease: the role of endothelial cells. Curr Top Dev Biol.

[12] Hinton RB, Lincoln J, Deutsch GH, Osinska H, Manning PB, Benson DW (2006). Extracellular matrix remodeling and organization in developing and diseased aortic valves. Circ Res.

[13] Vesely I (1998). The role of elastin in aortic valve mechanics. J Biomech.

[14] Grande-Allen KJ, Calabro A, Gupta V, Wight TN, Hascall VC, Vesely I (2004). Glycosaminoglycans and proteoglycans in normal mitral valve leaflets and chordae: association with regions of tensile and compressive loading. Glycobiology.

[15] Lincoln J, Alfieri CM, Yutzey KE (2006). BMP and FGF regulatory pathways control cell lineage diversification of heart valve precursor cells. Dev Biol.

[16] Syme DA, Gamperl K, Jones DR (2002). Delayed depolarization of the cog-wheel valve and pulmonary-to-systemic shunting in alligators. J Exp Biol.

[17] Amstrup Funder J, Christian Danielsen C, Baandrup U, Martin Bibby B, Carl Andelius T, Toft Brøndum E (2017). How heart valves evolve to adapt to an extreme-pressure system: morphologic and biomechanical properties of giraffe heart valves. J Heart Valve Dis.

[18] Horne TE, VandeKopple M, Sauls K, Koenig SN, Anstine LJ, Garg V (2015). Dynamic heterogeneity of the heart valve interstitial cell population in mitral valve health and disease. J Cardiovasc Dev Dis.

[19] Rabkin E, Aikawa M, Stone JR, Fukumoto Y, Libby P, Schoen FJ (2001). Activated interstitial myofibroblasts express catabolic enzymes and mediate matrix remodeling in myxomatous heart valves. Circulation.

[20] Rabkin-Aikawa E, Farber M, Aikawa M, Schoen FJ (2004). Dynamic and reversible changes of interstitial cell phenotype during remodeling of cardiac valves. J Heart Valve Dis.

[21] Helske S, Kupari M, Lindstedt KA, Kovanen PT (2007). Aortic valve stenosis: an active atheroinflammatory process. Curr Opin Lipidol.

[22] Anstine LJ, Bobba C, Ghadiali S, Lincoln J (2016). Growth and maturation of heart valves leads to changes in endothelial cell distribution, impaired function, decreased metabolism and reduced cell proliferation. J Mol Cell Cardiol.

[23] Bosse K, Hans CP, Zhao N, Koenig SN, Huang N, Guggilam A (2013). Endothelial nitric oxide signaling regulates Notch1 in aortic valve disease. J Mol Cell Cardiol.

[24] Huk DJ, Austin BF, Horne TE, Hinton RB, Ray WC, Heistad DD (2016). Valve endothelial cell-derived Tgfbeta1 signaling promotes nuclear localization of Sox9 in interstitial cells associated with attenuated calcification. Arterioscler Thromb Vasc Biol.

[25] Huk DJ, Austin BF, Horne TE, Hinton RB, Ray WC, Heistad DD, et al. Valve endothelial cell-derived Tgfbeta1 signaling promotes nuclear localization of Sox9 in interstitial cells associated with attenuated calcification. Arterioscler Thromb Vasc Biol 2015. 10.1161/ATVBAHA.115.306091 PMC4732913 26634652

[26] Laforest B, Andelfinger G, Nemer M (2011). Loss of Gata5 in mice leads to bicuspid aortic valve. J Clin Invest.

[27] MacGrogan D, D’Amato G, Travisano S, Martinez-Poveda B, Luxan G, Del Monte-Nieto G (2016). Sequential ligand-dependent Notch signaling activation regulates valve primordium formation and morphogenesis. Circ Res.

[28] Koenig SN, Bosse K, Majumdar U, Bonachea EM, Radtke F, Garg V (2016). Endothelial Notch1 is required for proper development of the semilunar valves and cardiac outflow tract. J Am Heart Assoc.

[29] Lincoln J, Alfieri CM, Yutzey KE (2004). Development of heart valve leaflets and supporting apparatus in chicken and mouse embryos. Dev Dyn.

[30] de Lange FJ, Moorman AF, Anderson RH, Manner J, Soufan AT, de Gier-de Vries C (2004). Lineage and morphogenetic analysis of the cardiac valves. Circ Res.

[31] Mercado-Pimentel ME, Runyan RB (2007). Multiple transforming growth factor-beta isoforms and receptors function during epithelial-mesenchymal cell transformation in the embryonic heart. Cells Tissues Organs.

[32] Nakajima Y, Yamagishi T, Hokari S, Nakamura H (2000). Mechanisms involved in valvuloseptal endocardial cushion formation in early cardiogenesis: roles of transforming growth factor (TGF)-beta and bone morphogenetic protein (BMP). Anat Rec.

[33] Person AD, Klewer SE, Runyan RB (2005). Cell biology of cardiac cushion development. Int Rev Cytol.

[34] Liebner S, Cattelino A, Gallini R, Rudini N, Iurlaro M, Piccolo S (2004). Beta-catenin is required for endothelial-mesenchymal transformation during heart cushion development in the mouse. J Cell Biol.

[35] Delot EC (2003). Control of endocardial cushion and cardiac valve maturation by BMP signaling pathways. Mol Genet Metab.

[36] Somi S, Buffing AA, Moorman AF, Van Den Hoff MJ (2004). Dynamic patterns of expression of BMP isoforms 2, 4, 5, 6, and 7 during chicken heart development. Anat Rec A Discov Mol Cell Evol Biol.

[37] Rivera-Feliciano J, Tabin CJ (2006). Bmp2 instructs cardiac progenitors to form the heart-valve-inducing field. Dev Biol.

[38] Ma L, Martin JF (2005). Generation of a Bmp2 conditional null allele. Genesis.

[39] Galvin KM, Donovan MJ, Lynch CA, Meyer RI, Paul RJ, Lorenz JN (2000). A role for smad6 in development and homeostasis of the cardiovascular system. Nat Genet.

[40] Matzuk MM, Lu N, Vogel H, Sellheyer K, Roop DR, Bradley A (1995). Multiple defects and perinatal death in mice deficient in follistatin. Nature.

[41] Brunet LJ, McMahon JA, McMahon AP, Harland RM (1998). Noggin, cartilage morphogenesis, and joint formation in the mammalian skeleton. Science.

[42] Bachiller D, Klingensmith J, Shneyder N, Tran U, Anderson R, Rossant J (2003). The role of chordin/Bmp signals in mammalian pharyngeal development and DiGeorge syndrome. Development.

[43] Ma L, Lu MF, Schwartz RJ, Martin JF (2005). Bmp2 is essential for cardiac cushion epithelial-mesenchymal transition and myocardial patterning. Development.

[44] Timmerman LA, Grego-Bessa J, Raya A, Bertran E, Perez-Pomares JM, Diez J (2004). Notch promotes epithelial-mesenchymal transition during cardiac development and oncogenic transformation. Genes Dev.

[45] Macgrogan D, Luna-Zurita L, de la Pompa JL (2011). Notch signaling in cardiac valve development and disease. Birth Defects Res A Clin Mol Teratol.

[46] Rutenberg JB, Fischer A, Jia H, Gessler M, Zhong TP, Mercola M (2006). Developmental patterning of the cardiac atrioventricular canal by Notch and hairy-related transcription factors. Development.

[47] Kokubo H, Tomita-Miyagawa S, Hamada Y, Saga Y (2007). Hesr1 and Hesr2 regulate atrioventricular boundary formation in the developing heart through the repression of Tbx2. Development.

[48] Venkatesh DA, Park KS, Harrington A, Miceli-Libby L, Yoon JK, Liaw L (2008). Cardiovascular and hematopoietic defects associated with Notch1 activation in embryonic Tie2-expressing populations. Circ Res.

[49] Luna-Zurita L, Prados B, Grego-Bessa J, Luxan G, del Monte G, Benguria A (2010). Integration of a Notch-dependent mesenchymal gene program and Bmp2-driven cell invasiveness regulates murine cardiac valve formation. J Clin Invest.

[50] Luxan G, D’Amato G, MacGrogan D, de la Pompa JL (2016). Endocardial Notch signaling in cardiac development and disease. Circ Res.

[51] Chang AC, Garside VC, Fournier M, Smrz J, Vrljicak P, Umlandt P (2014). A Notch-dependent transcriptional hierarchy promotes mesenchymal transdifferentiation in the cardiac cushion. Dev Dyn.

[52] Eisenberg L (1995). Medicine—molecular, monetary, or more than both?. JAMA.

[53] Sanford LP, Ormsby I, Gittenberger-de Groot AC, Sariola H, Friedman R, Boivin GP (1997). TGFbeta2 knockout mice have multiple developmental defects that are non-overlapping with other TGFbeta knockout phenotypes. Development.

[54] Bartram U, Molin DG, Wisse LJ, Mohamad A, Sanford LP, Doetschman T (2001). Double-outlet right ventricle and overriding tricuspid valve reflect disturbances of looping, myocardialization, endocardial cushion differentiation, and apoptosis in TGF-beta(2)-knockout mice. Circulation.

[55] Sridurongrit S, Larsson J, Schwartz R, Ruiz-Lozano P, Kaartinen V (2008). Signaling via the Tgf-beta type I receptor Alk5 in heart development. Dev Biol.

[56] Todorovic V, Finnegan E, Freyer L, Zilberberg L, Ota M, Rifkin DB (2011). Long form of latent TGF-beta binding protein 1 (Ltbp1L) regulates cardiac valve development. Dev Dyn.

[57] Lincoln J, Kist R, Scherer G, Yutzey KE (2007). Sox9 is required for precursor cell expansion and extracellular matrix organization during mouse heart valve development. Dev Biol.

[58] Wu B, Wang Y, Lui W, Langworthy M, Tompkins KL, Hatzopoulos AK (2011). Nfatc1 coordinates valve endocardial cell lineage development required for heart valve formation. Circ Res.

[59] Jiang X, Choudhary B, Merki E, Chien KR, Maxson RE, Sucov HM (2002). Normal fate and altered function of the cardiac neural crest cell lineage in retinoic acid receptor mutant embryos. Mech Dev.

[60] Jiang X, Rowitch DH, Soriano P, McMahon AP, Sucov HM (2000). Fate of the mammalian cardiac neural crest. Development.

[61] Verzi MP, McCulley DJ, De Val S, Dodou E, Black BL (2005). The right ventricle, outflow tract, and ventricular septum comprise a restricted expression domain within the secondary/anterior heart field. Dev Biol.

[62] Wessels A, van den Hoff MJ, Adamo RF, Phelps AL, Lockhart MM, Sauls K (2012). Epicardially derived fibroblasts preferentially contribute to the parietal leaflets of the atrioventricular valves in the murine heart. Dev Biol.

[63] Saxon JG, Baer DR, Barton JA, Hawkins T, Wu B, Trusk TC (2017). BMP2 expression in the endocardial lineage is required for AV endocardial cushion maturation and remodeling. Dev Biol.

[64] Azhar M, Brown K, Gard C, Chen H, Rajan S, Elliott DA (2011). Transforming growth factor Beta2 is required for valve remodeling during heart development. Dev Dyn.

[65] Cai X, Zhang W, Hu J, Zhang L, Sultana N, Wu B (2013). Tbx20 acts upstream of Wnt signaling to regulate endocardial cushion formation and valve remodeling during mouse cardiogenesis. Development.

[66] Amofa D, Hulin A, Nakada Y, Sadek HA, Yutzey KE. Hypoxia promotes primitive glycosaminoglycan-rich extracellular matrix composition in developing heart valves. Am J Physiol Heart Circ Physiol. (2017):ajpheart 00209 2017. 10.1152/ajpheart.00209.2017 PMC5814654 28842437

[67] Bowen CJ, Zhou J, Sung DC, Butcher JT (2015). Cadherin-11 coordinates cellular migration and extracellular matrix remodeling during aortic valve maturation. Dev Biol.

[68] Wilson CL, Gough PJ, Chang CA, Chan CK, Frey JM, Liu Y (2013). Endothelial deletion of ADAM17 in mice results in defective remodeling of the semilunar valves and cardiac dysfunction in adults. Mech Dev.

[69] Yu S, Crawford D, Tsuchihashi T, Behrens TW, Srivastava D (2011). The chemokine receptor CXCR7 functions to regulate cardiac valve remodeling. Dev Dyn.

[70] Yutzey KE (2017). Cardiomyocyte proliferation: teaching an old dogma new tricks. Circ Res.

[71] Anstine LJ, Horne TE, Horwitz EM, Lincoln J (2017). Contribution of extra-cardiac cells in murine heart valves is age-dependent. J Am Heart Assoc.

[72] Hajdu Z, Romeo SJ, Fleming PA, Markwald RR, Visconti RP, Drake CJ (2011). Recruitment of bone marrow-derived valve interstitial cells is a normal homeostatic process. J Mol Cell Cardiol.

[73] Visconti RP, Ebihara Y, LaRue AC, Fleming PA, McQuinn TC, Masuya M (2006). An in vivo analysis of hematopoietic stem cell potential: hematopoietic origin of cardiac valve interstitial cells. Circ Res.

[74] Bischoff J, Casanovas G, Wylie-Sears J, Kim DH, Bartko PE, Guerrero JL (2016). CD45 expression in mitral valve endothelial cells after myocardial infarction. Circ Res.

[75] Balaoing LR, Post AD, Liu H, Minn KT, Grande-Allen KJ (2014). Age-related changes in aortic valve hemostatic protein regulation. Arterioscler Thromb Vasc Biol.

[76] Stephens EH, Grande-Allen KJ (2007). Age-related changes in collagen synthesis and turnover in porcine heart valves. J Heart Valve Dis.

[77] Roger VL, Go AS, Lloyd-Jones DM, Benjamin EJ, Berry JD, Borden WB (2012). Heart disease and stroke statistics—2012 update: a report from the American Heart Association. Circulation.

[78] Mozaffarian D, Benjamin EJ, Go AS, Arnett DK, Blaha MJ, Cushman M (2016). Heart disease and stroke statistics—2016 update: a report from the American Heart Association. Circulation.

[79] Ginghina C, Florian A, Beladan C, Iancu M, Calin A, Popescu BA (2009). Calcific aortic valve disease and aortic atherosclerosis—two faces of the same disease?. Rom J Intern Med.

[80] Bonow RO, Leon MB, Doshi D, Moat N (2016). Management strategies and future challenges for aortic valve disease. Lancet.

[81] Chen JH, Simmons CA (2011). Cell-matrix interactions in the pathobiology of calcific aortic valve disease: critical roles for matricellular, matricrine, and matrix mechanics cues. Circ Res.

[82] Rajamannan NM (2010). Mechanisms of aortic valve calcification: the LDL-density-radius theory: a translation from cell signaling to physiology. Am J Physiol Heart Circ Physiol.

[83] Farrar EJ, Huntley GD, Butcher J (2015). Endothelial-derived oxidative stress drives myofibroblastic activation and calcification of the aortic valve. PLoS One.

[84] Towler DA (2008). Oxidation, inflammation, and aortic valve calcification peroxide paves an osteogenic path. J Am Coll Cardiol.

[85] Lee SH, Choi JH (2016). Involvement of immune cell network in aortic valve stenosis: communication between valvular interstitial cells and immune cells. Immune Netw.

[86] Rathan S, Yoganathan AP, O’Neill CW (2014). The role of inorganic pyrophosphate in aortic valve calcification. J Heart Valve Dis.

[87] Rutkovskiy A, Malashicheva A, Sullivan G, Bogdanova M, Kostareva A, Stenslokken KO (2017). Valve interstitial cells: the key to understanding the pathophysiology of heart valve calcification. J Am Heart Assoc.

[88] Yip CY, Chen JH, Zhao R, Simmons CA (2009). Calcification by valve interstitial cells is regulated by the stiffness of the extracellular matrix. Arterioscler Thromb Vasc Biol.

[89] Chen JH, Yip CY, Sone ED, Simmons CA (2009). Identification and characterization of aortic valve mesenchymal progenitor cells with robust osteogenic calcification potential. Am J Pathol.

[90] Rodriguez KJ, Masters KS (2009). Regulation of valvular interstitial cell calcification by components of the extracellular matrix. J Biomed Mater Res A.

[91] Benton JA, Kern HB, Anseth KS (2008). Substrate properties influence calcification in valvular interstitial cell culture. J Heart Valve Dis.

[92] He C, Tang H, Mei Z, Li N, Zeng Z, Darko KO (2017). Human interstitial cellular model in therapeutics of heart valve calcification. Amino Acids.

[93] Peacock JD, Huk DJ, Ediriweera HN, Lincoln J (2011). Sox9 transcriptionally represses Spp1 to prevent matrix mineralization in maturing heart valves and chondrocytes. PLoS One.

[94] Peacock JD, Levay AK, Gillaspie DB, Tao G, Lincoln J (2010). Reduced sox9 function promotes heart valve calcification phenotypes in vivo. Circ Res.

[95] Acharya A, Hans CP, Koenig SN, Nichols HA, Galindo CL, Garner HR (2011). Inhibitory role of Notch1 in calcific aortic valve disease. PLoS One.

[96] Tanaka K, Sata M, Fukuda D, Suematsu Y, Motomura N, Takamoto S (2005). Age-associated aortic stenosis in apolipoprotein E-deficient mice. J Am Coll Cardiol.

[97] Aikawa E, Nahrendorf M, Sosnovik D, Lok VM, Jaffer FA, Aikawa M (2007). Multimodality molecular imaging identifies proteolytic and osteogenic activities in early aortic valve disease. Circulation.

[98] Yoshioka M, Yuasa S, Matsumura K, Kimura K, Shiomi T, Kimura N (2006). Chondromodulin-I maintains cardiac valvular function by preventing angiogenesis. Nat Med.

[99] Barrick CJ, Roberts RB, Rojas M, Rajamannan NM, Suitt CB, O’Brien KD (2009). Reduced EGFR causes abnormal valvular differentiation leading to calcific aortic stenosis and left ventricular hypertrophy in C57BL/6J but not 129S1/SvImJ mice. Am J Physiol Heart Circ Physiol.

[100] Chen B, Bronson RT, Klaman LD, Hampton TG, Wang JF, Green PJ (2000). Mice mutant for Egfr and Shp2 have defective cardiac semilunar valvulogenesis. Nat Genet.

[101] Cheek JD, Wirrig EE, Alfieri CM, James JF, Yutzey KE (2012). Differential activation of valvulogenic, chondrogenic, and osteogenic pathways in mouse models of myxomatous and calcific aortic valve disease. J Mol Cell Cardiol.

[102] Weiss RM, Ohashi M, Miller JD, Young SG, Heistad DD (2006). Calcific aortic valve stenosis in old hypercholesterolemic mice. Circulation.

[103] Nigam V, Srivastava D (2009). Notch1 represses osteogenic pathways in aortic valve cells. J Mol Cell Cardiol.

[104] Nus M, MacGrogan D, Martinez-Poveda B, Benito Y, Casanova JC, Fernandez-Aviles F (2011). Diet-induced aortic valve disease in mice haploinsufficient for the Notch pathway effector RBPJK/CSL. Arterioscler Thromb Vasc Biol.

[105] Li Z, Feng L, Wang CM, Zheng QJ, Zhao BJ, Yi W (2012). Deletion of RBP-J in adult mice leads to the onset of aortic valve degenerative diseases. Mol Biol Rep.

[106] Huk DJ, Hammond HL, Kegechika H, Lincoln J (2013). Increased dietary intake of vitamin A promotes aortic valve calcification in vivo. Arterioscler Thromb Vasc Biol.

[107] Schmidt N, Brandsch C, Kuhne H, Thiele A, Hirche F, Stangl GI (2012). Vitamin D receptor deficiency and low vitamin D diet stimulate aortic calcification and osteogenic key factor expression in mice. PLoS One.

[108] Rajamannan NM, Evans FJ, Aikawa E, Grande-Allen KJ, Demer LL, Heistad DD (2011). Calcific aortic valve disease: not simply a degenerative process: a review and agenda for research from the National Heart and Lung and Blood Institute Aortic Stenosis Working Group. Executive summary: calcific aortic valve disease-2011 update. Circulation.

[109] Lincoln J, Lange AW, Yutzey KE (2006). Hearts and bones: shared regulatory mechanisms in heart valve, cartilage, tendon, and bone development. Dev Biol.

[110] Bertazzo S, Gentleman E (2017). Aortic valve calcification: a bone of contention. Eur Heart J.

[111] Walker GA, Masters KS, Shah DN, Anseth KS, Leinwand LA (2004). Valvular myofibroblast activation by transforming growth factor-beta: implications for pathological extracellular matrix remodeling in heart valve disease. Circ Res.

[112] Clark-Greuel JN, Connolly JM, Sorichillo E, Narula NR, Rapoport HS, Mohler ER (2007). Transforming growth factor-beta1 mechanisms in aortic valve calcification: increased alkaline phosphatase and related events. Ann Thorac Surg.

[113] Jian B, Narula N, Li QY, Mohler ER, Levy RJ (2003). Progression of aortic valve stenosis: TGF-beta1 is present in calcified aortic valve cusps and promotes aortic valve interstitial cell calcification via apoptosis. Ann Thorac Surg.

[114] Kulkarni AB, Karlsson S (1993). Transforming growth factor-beta 1 knockout mice. A mutation in one cytokine gene causes a dramatic inflammatory disease. Am J Pathol.

[115] Kaartinen V, Voncken JW, Shuler C, Warburton D, Bu D, Heisterkamp N (1995). Abnormal lung development and cleft palate in mice lacking TGF-beta 3 indicates defects of epithelial-mesenchymal interaction. Nat Genet.

[116] Proetzel G, Pawlowski SA, Wiles MV, Yin M, Boivin GP, Howles PN (1995). Transforming growth factor-beta 3 is required for secondary palate fusion. Nat Genet.

[117] Wu M, Chen G, Li YP (2016). TGF-beta and BMP signaling in osteoblast, skeletal development, and bone formation, homeostasis and disease. Bone Res.

[118] Huang Z, Ren PG, Ma T, Smith RL, Goodman SB (2010). Modulating osteogenesis of mesenchymal stem cells by modifying growth factor availability. Cytokine.

[119] Noel D, Gazit D, Bouquet C, Apparailly F, Bony C, Plence P (2004). Short-term BMP-2 expression is sufficient for in vivo osteochondral differentiation of mesenchymal stem cells. Stem Cells.

[120] Gu K, Zhang L, Jin T, Rutherford RB (2004). Identification of potential modifiers of Runx2/Cbfa1 activity in C2C12 cells in response to bone morphogenetic protein-7. Cells Tissues Organs.

[121] Shen B, Wei A, Whittaker S, Williams LA, Tao H, Ma DD (2010). The role of BMP-7 in chondrogenic and osteogenic differentiation of human bone marrow multipotent mesenchymal stromal cells in vitro. J Cell Biochem.

[122] Medici D, Shore EM, Lounev VY, Kaplan FS, Kalluri R, Olsen BR (2010). Conversion of vascular endothelial cells into multipotent stem-like cells. Nat Med.

[123] van Dinther M, Visser N, de Gorter DJ, Doorn J, Goumans MJ, de Boer J (2010). ALK2 R206H mutation linked to fibrodysplasia ossificans progressiva confers constitutive activity to the BMP type I receptor and sensitizes mesenchymal cells to BMP-induced osteoblast differentiation and bone formation. J Bone Miner Res.

[124] Yang C, Yang L, Wan M, Cao X (2010). Generation of a mouse model with expression of bone morphogenetic protein type II receptor lacking the cytoplasmic domain in osteoblasts. Ann N Y Acad Sci.

[125] Mishina Y, Starbuck MW, Gentile MA, Fukuda T, Kasparcova V, Seedor JG (2004). Bone morphogenetic protein type IA receptor signaling regulates postnatal osteoblast function and bone remodeling. J Biol Chem.

[126] Kamiya N, Kobayashi T, Mochida Y, Yu PB, Yamauchi M, Kronenberg HM (2010). Wnt inhibitors Dkk1 and Sost are downstream targets of BMP signaling through the type IA receptor (BMPRIA) in osteoblasts. J Bone Miner Res.

[127] Kamiya N, Ye L, Kobayashi T, Lucas DJ, Mochida Y, Yamauchi M (2008). Disruption of BMP signaling in osteoblasts through type IA receptor (BMPRIA) increases bone mass. J Bone Miner Res.

[128] Kamiya N, Ye L, Kobayashi T, Mochida Y, Yamauchi M, Kronenberg HM (2008). BMP signaling negatively regulates bone mass through sclerostin by inhibiting the canonical Wnt pathway. Development.

[129] Danielian PS, Muccino D, Rowitch DH, Michael SK, AP MM (1998). Modification of gene activity in mouse embryos in utero by a tamoxifen-inducible form of Cre recombinase. Curr Biol.

[130] Monzack EL, Masters KS (2011). Can valvular interstitial cells become true osteoblasts? A side-by-side comparison. J Heart Valve Dis.

[131] Wirrig EE, Gomez MV, Hinton RB, Yutzey KE (2015). COX2 inhibition reduces aortic valve calcification in vivo. Arterioscler Thromb Vasc Biol.

[132] Thomas PS, Sridurongrit S, Ruiz-Lozano P, Kaartinen V (2012). Deficient signaling via Alk2 (Acvr1) leads to bicuspid aortic valve development. PLoS One.

[133] Caira FC, Stock SR, Gleason TG, McGee EC, Huang J, Bonow RO (2006). Human degenerative valve disease is associated with up-regulation of low-density lipoprotein receptor-related protein 5 receptor-mediated bone formation. J Am Coll Cardiol.

[134] Miller JD, Weiss RM, Serrano KM, Castaneda LE, Brooks RM, Zimmerman K (2010). Evidence for active regulation of pro-osteogenic signaling in advanced aortic valve disease. Arterioscler Thromb Vasc Biol.

[135] Askevold ET, Gullestad L, Aakhus S, Ranheim T, Tonnessen T, Solberg OG (2012). Secreted Wnt modulators in symptomatic aortic stenosis. J Am Heart Assoc.

[136] Gu GJ, Chen T, Zhou HM, Sun KX, Li J (2014). Role of Wnt/beta-catenin signaling pathway in the mechanism of calcification of aortic valve. J Huazhong Univ Sci Technol Med Sci.

[137] Gao X, Zhang L, Gu G, Wu PH, Jin S, Hu W (2015). The effect of oxLDL on aortic valve calcification via the Wnt/ beta-catenin signaling pathway: an important molecular mechanism. J Heart Valve Dis.

[138] Albanese I, Yu B, Al-Kindi H, Barratt B, Ott L, Al-Refai M (2017). Role of noncanonical Wnt signaling pathway in human aortic valve calcification. Arterioscler Thromb Vasc Biol.

[139] Gaur T, Lengner CJ, Hovhannisyan H, Bhat RA, Bodine PV, Komm BS (2005). Canonical WNT signaling promotes osteogenesis by directly stimulating Runx2 gene expression. J Biol Chem.

[140] Kratchmarova I, Blagoev B, Haack-Sorensen M, Kassem M, Mann M (2005). Mechanism of divergent growth factor effects in mesenchymal stem cell differentiation. Science.

[141] Cai T, Sun D, Duan Y, Wen P, Dai C, Yang J (2016). WNT/beta-catenin signaling promotes VSMCs to osteogenic transdifferentiation and calcification through directly modulating Runx2 gene expression. Exp Cell Res.

[142] White MP, Theodoris CV, Liu L, Collins WJ, Blue KW, Lee JH (2015). NOTCH1 regulates matrix gla protein and calcification gene networks in human valve endothelium. J Mol Cell Cardiol.

[143] Hofmann JJ, Briot A, Enciso J, Zovein AC, Ren S, Zhang ZW (2012). Endothelial deletion of murine Jag1 leads to valve calcification and congenital heart defects associated with Alagille syndrome. Development.

[144] Choi KY, Kim HJ, Lee MH, Kwon TG, Nah HD, Furuichi T (2005). Runx2 regulates FGF2-induced Bmp2 expression during cranial bone development. Dev Dyn.

[145] Tezuka K, Yasuda M, Watanabe N, Morimura N, Kuroda K, Miyatani S (2002). Stimulation of osteoblastic cell differentiation by Notch. J Bone Miner Res.

[146] Nobta M, Tsukazaki T, Shibata Y, Xin C, Moriishi T, Sakano S (2005). Critical regulation of bone morphogenetic protein-induced osteoblastic differentiation by Delta1/Jagged1-activated Notch1 signaling. J Biol Chem.

[147] de Jong DS, Steegenga WT, Hendriks JM, van Zoelen EJ, Olijve W, Dechering KJ (2004). Regulation of Notch signaling genes during BMP2-induced differentiation of osteoblast precursor cells. Biochem Biophys Res Commun.

[148] Bi W, Deng JM, Zhang Z, Behringer RR, de Crombrugghe B (1999). Sox9 is required for cartilage formation. Nat Genet.

[149] Bi W, Huang W, Whitworth DJ, Deng JM, Zhang Z, Behringer RR (2001). Haploinsufficiency of Sox9 results in defective cartilage primordia and premature skeletal mineralization. Proc Natl Acad Sci U S A.

[150] Zhou G, Zheng Q, Engin F, Munivez E, Chen Y, Sebald E (2006). Dominance of SOX9 function over RUNX2 during skeletogenesis. Proc Natl Acad Sci U S A.

[151] Cheng A, Genever PG (2010). SOX9 determines RUNX2 transactivity by directing intracellular degradation. J Bone Miner Res.

[152] Jansen F, Xiang X, Werner N (2017). Role and function of extracellular vesicles in calcific aortic valve disease. Eur Heart J.

[153] Krohn JB, Hutcheson JD, Martinez-Martinez E, Aikawa E (2016). Extracellular vesicles in cardiovascular calcification: expanding current paradigms. J Physiol.

[154] Goettsch C, Hutcheson JD, Aikawa M, Iwata H, Pham T, Nykjaer A (2016). Sortilin mediates vascular calcification via its recruitment into extracellular vesicles. J Clin Investig.

[155] Arjunon S, Rathan S, Jo H, Yoganathan AP (2013). Aortic valve: mechanical environment and mechanobiology. Ann Biomed Eng.

[156] Simmons CA, Grant GR, Manduchi E, Davies PF (2005). Spatial heterogeneity of endothelial phenotypes correlates with side-specific vulnerability to calcification in normal porcine aortic valves. Circ Res.

[157] Moore BL, Dasi LP (2015). Coronary flow impacts aortic leaflet mechanics and aortic sinus hemodynamics. Ann Biomed Eng.

[158] Fisher CI, Chen J, Merryman WD (2013). Calcific nodule morphogenesis by heart valve interstitial cells is strain dependent. Biomech Model Mechanobiol.

[159] Arzani A, Mofrad MRK (2017). A strain-based finite element model for calcification progression in aortic valves. J Biomech.

[160] Nkomo VT, Gardin JM, Skelton TN, Gottdiener JS, Scott CG, Enriquez-Sarano M (2006). Burden of valvular heart diseases: a population-based study. Lancet.

[161] Iung B, Vahanian A (2011). Epidemiology of valvular heart disease in the adult. Nat Rev Cardiol.

[162] Lindroos M, Kupari M, Heikkila J, Tilvis R (1993). Prevalence of aortic valve abnormalities in the elderly: an echocardiographic study of a random population sample. J Am Coll Cardiol.

[163] Bonow RO (2013). Chronic mitral regurgitation and aortic regurgitation: have indications for surgery changed?. J Am Coll Cardiol.

[164] Mohr FW, Holzhey D, Mollmann H, Beckmann A, Veit C, Figulla HR (2014). The German aortic valve registry: 1-year results from 13,680 patients with aortic valve disease. Eur J Cardiothorac Surg.

[165] Thourani VH, Suri RM, Gunter RL, Sheng S, O’Brien SM, Ailawadi G (2015). Contemporary real-world outcomes of surgical aortic valve replacement in 141,905 low-risk, intermediate-risk, and high-risk patients. Ann Thorac Surg.

[166] Barreto-Filho JA, Wang Y, Dodson JA, Desai MM, Sugeng L, Geirsson A (2013). Trends in aortic valve replacement for elderly patients in the United States, 1999-2011. JAMA.

[167] Brown JM, O’Brien SM, Wu C, Sikora JA, Griffith BP, Gammie JS (2009). Isolated aortic valve replacement in North America comprising 108,687 patients in 10 years: changes in risks, valve types, and outcomes in the Society of Thoracic Surgeons National Database. J Thorac Cardiovasc Surg.

[168] Antonini-Canterin F, Hirsu M, Popescu BA, Leiballi E, Piazza R, Pavan D (2008). Stage-related effect of statin treatment on the progression of aortic valve sclerosis and stenosis. Am J Cardiol.

[169] Aronow WS, Ahn C, Kronzon I, Goldman ME (2001). Association of coronary risk factors and use of statins with progression of mild valvular aortic stenosis in older persons. Am J Cardiol.

[170] Moura LM, Ramos SF, Zamorano JL, Barros IM, Azevedo LF, Rocha-Goncalves F (2007). Rosuvastatin affecting aortic valve endothelium to slow the progression of aortic stenosis. J Am Coll Cardiol.

[171] Novaro GM, Tiong IY, Pearce GL, Lauer MS, Sprecher DL, Griffin BP (2001). Effect of hydroxymethylglutaryl coenzyme a reductase inhibitors on the progression of calcific aortic stenosis. Circulation.

[172] Rosenhek R, Rader F, Loho N, Gabriel H, Heger M, Klaar U (2004). Statins but not angiotensin-converting enzyme inhibitors delay progression of aortic stenosis. Circulation.

[173] Shavelle DM, Takasu J, Budoff MJ, Mao S, Zhao XQ, O’Brien KD (2002). HMG CoA reductase inhibitor (statin) and aortic valve calcium. Lancet.

[174] Bellamy MF, Pellikka PA, Klarich KW, Tajik AJ, Enriquez-Sarano M (2002). Association of cholesterol levels, hydroxymethylglutaryl coenzyme-A reductase inhibitor treatment, and progression of aortic stenosis in the community. J Am Coll Cardiol.

[175] Chan KL, Teo K, Dumesnil JG, Ni A, Tam J, ASTRONOMER Investigators (2010). Effect of lipid lowering with rosuvastatin on progression of aortic stenosis: results of the aortic stenosis progression observation: measuring effects of rosuvastatin (ASTRONOMER) trial. Circulation.

[176] Dichtl W, Alber HF, Feuchtner GM, Hintringer F, Reinthaler M, Bartel T (2008). Prognosis and risk factors in patients with asymptomatic aortic stenosis and their modulation by atorvastatin (20 mg). Am J Cardiol.

[177] Cowell SJ, Newby DE, Prescott RJ, Bloomfield P, Reid J, Northridge DB (2005). A randomized trial of intensive lipid-lowering therapy in calcific aortic stenosis. New Engl J Med.

[178] Mohler ER, Wang H, Medenilla E, Scott C (2007). Effect of statin treatment on aortic valve and coronary artery calcification. J Heart Valve Dis.

[179] Panahi Y, Sahebkar A, Taghipour HR, Dadjou Y, Pishgoo B, Rakhshankhah AS (2013). Atorvastatin therapy is not associated with slowing the progression of aortic stenosis: findings of a randomized controlled trial. Clin Lab.

[180] Pohle K, Maffert R, Ropers D, Moshage W, Stilianakis N, Daniel WG (2001). Progression of aortic valve calcification: association with coronary atherosclerosis and cardiovascular risk factors. Circulation.

[181] Rossebo AB, Pedersen TR, Boman K, Brudi P, Chambers JB, Egstrup K (2008). Intensive lipid lowering with simvastatin and ezetimibe in aortic stenosis. New Engl J Med.

